# 30-day outcomes for self-gripping mesh versus non-self-gripping mesh in minimally invasive extraperitoneal ventral hernia repair

**DOI:** 10.1007/s00464-026-13116-6

**Published:** 2026-07-15

**Authors:** Ayesha Siddiq, Nicolette M. Winder, Cassandra Hennessy, Jeffrey Marks, Joshua L. Lyons, Samuel J. Zolin

**Affiliations:** 1 University Hospitals Cleveland Medical Center, 11100 Euclid Ave, Cleveland, OH 44106 USA; 2 Case Western Reserve University, Cleveland, OH USA; 3 Department of Biostatistics, Vanderbilt University Medical Center, Nashville, TN USA

**Keywords:** Ventral hernia repair, Minimally invasive hernia repair, Self-gripping mesh, Robotic hernia repair, Hernia mesh

## Abstract

**Background:**

Prior work suggests that use of self-gripping mesh (SGM) may be associated with decreased wound complications and pain compared to non-self-gripping mesh (NSGM) after open ventral hernia repair (VHR). Outcomes following minimally invasive (MIS) extraperitoneal VHR are unknown. We hypothesized that use of SGM in MIS extraperitoneal VHR would be associated with decreased wound complications and postoperative pain.

**Methods:**

A retrospective cohort study using prospective data from the Abdominal Core Health Quality Collaborative was performed. Adults undergoing clean, elective, MIS retromuscular and/or preperitoneal VHR using permanent synthetic SGM or NSGM were included. Cases with a second mesh, barrier-coated mesh, or anatomic inguinal mesh or without a 30-day follow-up were excluded. 1:1 propensity score matching (PSM) was utilized to generate comparable groups. The primary outcome was 30-day surgical site infection (SSI). Secondary outcomes included surgical site occurrences (SSO), SSO/I requiring procedural intervention (SSOPI), operative time, pain and hernia-related quality of life (HerQLes) at 30 d and 1 year, and hernia recurrence at 1–5 years.

**Results:**

After PSM, 1378 patients remained (689 SGM, 689 NSGM). Baseline covariates were well-matched. Operative time was longer for SGM compared to NSGM cases (*p* = 0.008). Thirty-day SSI, SSO, and SSOPI were rare and comparable between groups. There was no difference in pain or HerQLes scores at 30-day or 1-year follow-up. Hernia recurrence rates were comparable at 1 year; however, SGM had significantly higher rates of hernia recurrence at 2–4 years.

**Conclusions:**

There were no observed clinical advantages to use of polyester-based SGM in MIS extraperitoneal VHR, with a concerning trend toward increased long-term hernia recurrence. Further prospective evaluation and long-term surveillance are needed to define the role of SGM in MIS extraperitoneal VHR.

Over the past decade, minimally invasive surgery (MIS) has become an increasingly common approach for ventral hernia repair (VHR). Advancements in and access to robotic platforms as well as enhanced surgeon proficiency have enabled the performance of procedures that were traditionally conducted via open surgery, including complex techniques such as component separation [1]. With the proliferation of robotic technology, there has been a notable shift toward extraperitoneal approaches, such as preperitoneal and retromuscular (retrorectus) techniques [2].

Concurrent with these technical developments, options for mesh materials have evolved. Since its introduction in 2006, self-gripping mesh (SGM), featuring resorbable microgrips that adhere to surrounding tissue, has emerged as a potential alternative to traditional non-self-gripping mesh (NSGM) [3]. The purported benefits of SGM include reductions in fixation-related trauma, operative time, and postoperative pain. Prior study in open VHR has suggested that SGM may be associated with lower rates of wound complications and decreased pain compared to NSGM when used in the retromuscular space [4, 5]. Conversely, other analyses have shown higher overall complication rates and an increased frequency of reinterventions with SGM compared to traditional fixation techniques [6]. As such, the clinical benefits of SGM remain uncertain in the context of ventral hernia repair.

Comparative outcomes of SGM and NSGM in the context of MIS extraperitoneal VHR remain unexplored. Given the differences in baseline probability of wound morbidity, tissue tension during fascial closure, and mesh handling between open and MIS approaches, the performance and complication profiles of SGM in the minimally invasive setting may differ significantly from those observed in open surgery.

In this study, we aim to evaluate 30-day postoperative outcomes following MIS retrorectus and preperitoneal VHR, comparing SGM versus NSGM. We hypothesize that the use of SGM will be associated with decreased wound complications and reduced postoperative pain relative to NSGM in the context of MIS extraperitoneal VHR.

## Materials and methods

We conducted a retrospective cohort study utilizing prospectively collected data from the Abdominal Core Health Quality Collaborative (ACHQC) database. The ACHQC is an international registry designed to facilitate continuous quality improvement and outcomes of research in abdominal wall reconstruction, featuring data from more than 160,000 patients entered by over 500 participating surgeons [7]. Adult patients (≥ 18 years) who underwent VHR with a clean wound class via an MIS (laparoscopic or robotic) extraperitoneal approach (either retrorectus and/or preperitoneal) with uncoated, permanent synthetic mesh between the inception of the registry and July 2024 were included. Only patients with documented mesh type and size were included. Meshes that were utilized in this cohort were reviewed and classified as either SGM or NSGM. Of note, only polyester-based SGM were available during this time period.

Cases involving the use of a second mesh, barrier-coated mesh, anatomic inguinal mesh, or missing mesh characteristics were excluded, as were cases without 30-day follow-up. Cases with concomitant procedures to repair other hernias (e.g., inguinal hernias) or with additional procedures that would result in a non-clean wound class (e.g., cholecystectomy) were also excluded. Relevant past medical and surgical history, as well as technical aspects of the operation, such as mesh fixation and drain use, and postoperative clinical and patient-reported outcomes were abstracted from the ACHQC.

To adjust for potential confounding due to the observational nature of the study, we used propensity score matching to create comparable groups based on demographic and clinical variables, including age, BMI, smoking status, COPD, ASA classification, hernia width, hernia length, EHS hernia location classification (e.g., M2, L2, etc.), mesh location (e.g., only retromuscular, only preperitoneal, or both), use of myofascial release, recurrent vs. primary hernia, and drain usage. Multiple imputation using predictive mean matching was used to impute missing variables before matching. Propensity scores were estimated using logistic regression in a 1:1 nearest neighbor’s fashion.

To investigate the set of circumstances in which SGM might provide the theoretical greatest reduction in pain, a second propensity score match was performed to generate groups of patients that either had a repair with SGM without additional fixation or with NSGM with fixation.

Data access, cleaning, and statistical analysis were performed by an ACHQC-affiliated biostatistician (CH) using *R* (version 4.5.1). Wilcoxon rank-sum tests were used to compare continuous variables, and Pearson’s chi-squared tests were used to compare categorical variables. A *p* value of < 0.05 was considered statistically significant. Given that this was a retrospective cohort study, no pre-specified power analysis was performed.

The primary outcome was 30-day surgical site infection (SSI). Secondary outcomes included other short-term complications such as 30-day surgical site occurrences (SSO), SSI or SSO requiring procedural intervention (SSOPI), as well as operative time and reoperation at 30 d and 1 year. Hernia recurrence at 30-day follow-up and up to 5 years after surgery was also evaluated and this was classified in three ways. Patient-reported recurrence was defined as a positive response to the Hernia Recurrence Inventory (HRI) [8]. Surgeon-reported recurrence was defined as a recurrence identified either on physical exam or through imaging. Pragmatic recurrence incorporates both patient-reported and surgeon-reported recurrence to provide a measure of recurrence when one or the other is missing, as defined in a previous randomized controlled trial [9]. As an example, a positive response to the HRI without concurrent clinical follow-up was considered to be a pragmatic recurrence. However, a positive response to the HRI with a CT scan or surgeon exam demonstrating no hernia recurrence (e.g., negative clinical recurrence) would be considered negative with regard to pragmatic recurrence. Of note, imputation was used with pragmatic recurrence to address missing data. This was done in a retrograde fashion and only with negative measures of recurrence. For instance, if a patient was missing follow-up at 1 year but was negative for recurrence at 2 years, they would be considered to have a negative pragmatic recurrence at 1 year. There was no forward imputation of recurrence, nor were positive pragmatic recurrences imputed backwards, as it would not be possible to distinguish when a recurrence occurred.

Patient-reported outcomes were also assessed at baseline, 30 d, and 1 year. Pain was measured by the patient-reported outcomes measurement information system (PROMIS) Pain Intensity score [10]. A higher PROMIS score indicates a greater level of pain; the minimum clinically significant difference (MCID) was defined as a range from 2.0 to 3.0 points [11]. Hernia-specific quality of life was measured using the HerQLes survey, which consists of 12 items and is scored from 0 to 100 [12]. A higher HerQLes score indicates a better quality of life. The MCID of HerQLes has been defined as a difference of at least 15.6 points [13].

Institutional Review Board (IRB) approval was obtained for this study, and all data were collected in compliance with ethical research standards. Given that no identifying information was utilized, consent was not required from study participants. Data reporting followed Reporting of studies Conducted using Observational Routinely Collected Health Data (RECORD) guidelines. [14].

## Results

**Fig. 1:**
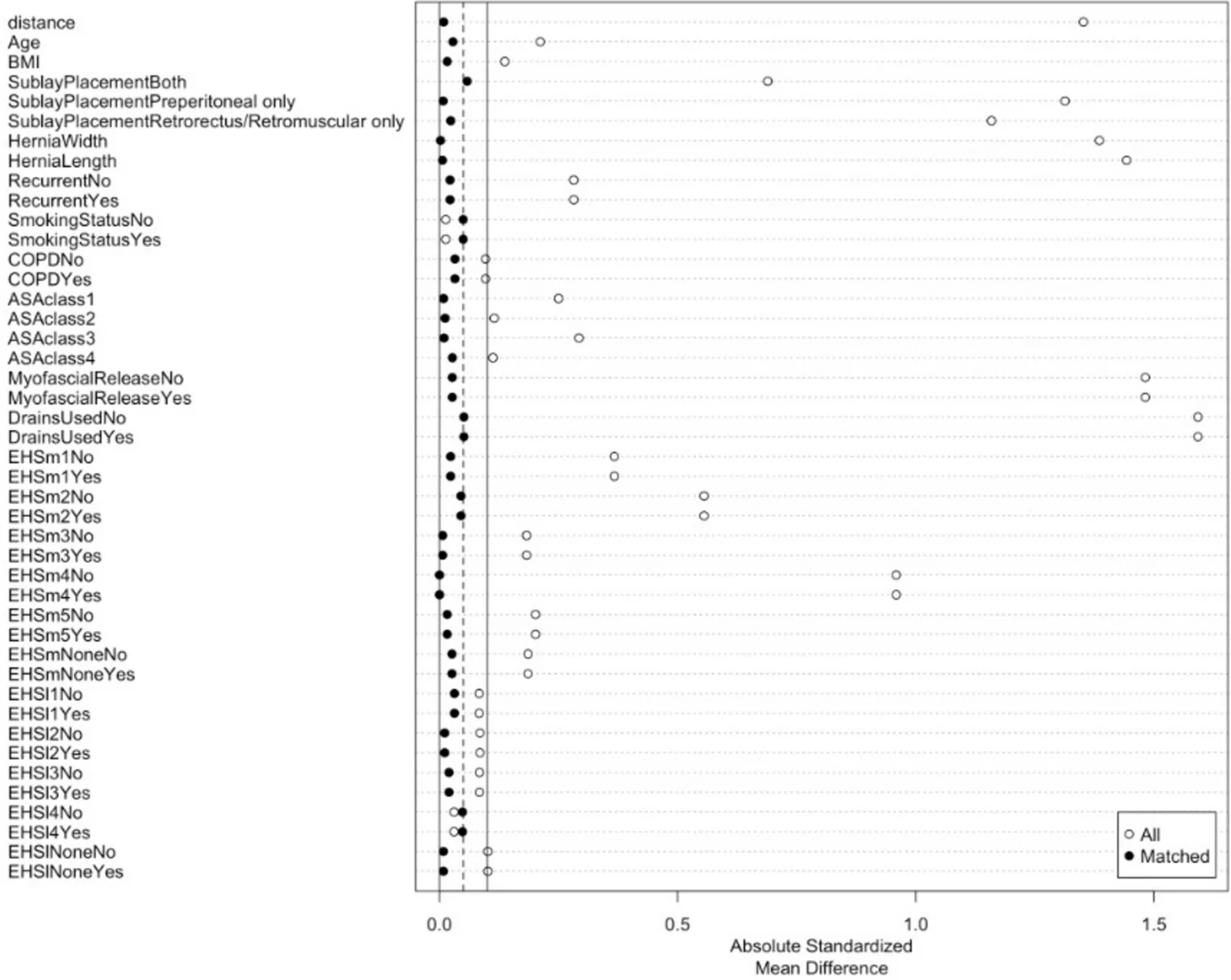
Standardized mean differences of baseline covariates before and after 1:1 propensity score matching for patients undergoing minimally invasive extraperitoneal ventral hernia repair with self-gripping mesh versus non-self-gripping mesh. After matching, all covariates demonstrated improved and adequate balance with absolute standardized mean differences < 0.1

A total of 4827 patients in the ACHQC met inclusion criteria. Before matching, there were substantial differences in baseline medical, operative, and hernia characteristics between patients in the SGM versus NSGM cohort. After 1:1 propensity score matching, 1378 patients remained (689 SGM and 689 NSGM), and baseline covariates were well balanced with a standardized mean difference of less than 0.1 (Fig. 1). There were no significant differences in baseline demographic, clinical, and hernia characteristics between both groups following matching (Tables 1 and 2).

**Table 1:** Patient demographic characteristics after propensity score matchingª

Characteristic	NSGM (*n* = 689)	SGM (*n* = 689)	*p* value
Age, years	53 ± 14	54 ± 14	0.9
BMI, kg/m^2^	32 ± 6	32 ± 7	0.8
Sex			0.5
Male	409 (59)	397 (58)	
Female	280 (41)	292 (42)	
Race			0.4
White	527 (76)	540 (78)	
Non-white	162 (24)	149 (22)	
Hypertension	305 (44)	264 (38)	0.02
Diabetes mellitus	74 (11)	91 (13)	0.2
COPD	27 (4)	23 (3)	0.6
Smoking (current within one month)	75 (11)	65 (9)	0.4
Immunosuppression	14 (2)	18 (3)	0.5
ASA Class			0.9
1	94 (14)	92 (13)	
2	374 (54)	378 (55)	
3	220 (32)	217 (31)	
4	1 (0)	2 (0)	

ªData are presented as mean ± SD or number (percentage). *BMI *Body mass index. *COPD* Chronic obstructive pulmonary disease. *ASA* American Society of Anesthesiologists

**Table 2:** Hernia-specific historyª

Characteristic	NSGM (*n* = 689)	SGM (*n* = 689)	*p* value
Recurrent hernia	80 (12)	85 (12)	0.7
Number of prior repairs			0.9
0	609 (89)	604 (88)	
1	64 (9)	72 (10)	
2	11 (2)	10 (1)	
3 +	4 (0)	3 (0)	
Midline hernia	586 (85)	581 (84)	0.1
Lateral hernia	92 (13)	83 (12)	0.1
Both midline and lateral hernia	9 (1)	20 (3)	0.1
Hernia width, cm	3 ± 2	3 ± 2	0.3
Hernia length, cm	4 ± 3	4 ± 4	0.6

ªData are presented as mean ± SD or number (percentage)

Operative details are demonstrated in Table 3. The vast majority of patients underwent fascial closure via a running approach. Absorbable suture was used more commonly than permanent suture, with no significant difference in rates of use of each between groups. Mesh suture was recorded as having been used for 5 patients in the SGM group, compared to 0 patients in the NSGM group (*p* = 0.03). Repair plane (preperitoneal, retromuscular, or both), use of myofascial release, and drain placement were similar between groups. Mesh fixation was utilized less often in the SGM group compared with the NSGM group (23 vs 82%). Among cases utilizing fixation, sutures were the most common method in both groups (74 vs 89%). Absorbable fixation sutures were used more often in the NSGM group (94 vs 68%), while permanent sutures were more commonly used in the SGM group (8 vs 4%). Over 80% of cases in both groups utilized the preperitoneal plane, with a smaller proportion utilizing the retrorectus plane. Among patients undergoing component separation, there was a slightly higher rate of transversus abdominis release in the SGM group (19 vs 15%), though this was not statistically significant. The extent of craniocaudal mesh overlap was similar in both groups (*p* = 0.5); however, the extent of lateral mesh overlap was greater in the NSGM group (*p* < 0.001). Operative time differed between groups, with SGM associated with longer operative duration overall (*p* = 0.008), though the proportion of cases that took more than 2 h was similar between groups (*p* = 0.19).

**Table 3:** Operative detailsª

	NSGM (*n* = 689)	SGM (*n* = 689)	*p* value
Operative approach			0.1
Laparoscopic	38 (6)	26 (4)	
Robotic	651 (94)	663 (96)	
Fascial closure	660 (96)	662 (97)	0.6
Fascial closure technique			
Running	636 (96)	645 (97)	0.4
Figure-of-eight	19 (3)	10 (2)	0.09
Simple interrupted	6 (1)	11 (2)	0.2
Mattress	1 (0)	1 (0)	1.00
Fascial closure material			
Absorbable suture	549 (83)	562 (86)	0.4
Permanent suture	114 (17)	100 (15)	0.3
Mesh suture	0 (0)	5 (1)	0.03
Preperitoneal sublay placement	581 (84)	575 (83)	0.7
Retrorectus/retromuscular sublay placement	119 (17)	121 (18)	0.9
Mesh length, cm	15 ± 7	15 ± 6	0.4
Mesh width, cm	12 ± 4	11 ± 3	< 0.001
Mesh craniocaudal overlap, cm	6 ± 3	5 ± 2	0.5
Mesh lateral overlap, cm	5 ± 2	4 ± 1	< 0.001
Mesh fixation	568 (82)	157 (23)	< 0.001
Sutures	519 (89)	147 (74)	< 0.001
Absorbable	502 (94)	131 (68)	< 0.001
Permanent	19 (4)	16 (8)	0.008
Staples	4 (1)	0 (0)	0.3
Tacks	39 (7)	8 (4)	0.2
Adhesives	17 (3)	2 (1)	0.1
Myofascial release	77 (11)	83 (12)	0.6
Transversus abdominis	21 (15)	24 (19)	0.5
Posterior rectus sheath incision	74 (54)	71 (55)	0.9
Drain placed	13 (2)	9 (1)	0.4
Operative time			0.008
0–59 min	124 (18)	107 (16)	
60–119 min	382 (55)	377 (55)	
120–179 min	124 (18)	130 (19)	
180–239 min	48 (7)	41 (6)	
≥ 240 min	11 (2)	34 (5)	

ªData are presented as mean ± SD or number (percentage)

Post-operative clinical outcomes are shown in Table 4. The primary outcome, 30-day SSI, was rare, occurring in only 2 cases each within SGM and NSGM groups (*p* = 1). Rates of SSO and SSOPI were similarly low and not statistically significant (*p* = 0.6 and 0.4, respectively). At 1 year, SSI and SSOPI remained rare and comparable between both groups (0 vs 0%). Although SSO rates were numerically higher in the SGM group among patients with 1-year clinical follow-up (6 vs 0%, *p* = 0.2), this difference was not statistically significant (Table 4).

**Table 4:** Post-operative outcomes

Outcome	*N*	NSGM (*n*=689)	SGM (*n*=689)	*p* value
At 30-day follow-up				
SSO	1378	35 (5)	40 (6)	0.6
SSI	1378	2 (0)	2 (0)	1
SSO/I requiring procedural intervention	1378	7 (1)	4 (1)	0.4
Reoperation	1377	3 (0)	5 (1)	0.5
At 1-year follow-up				
SSO	46	0 (0)	1 (6)	0.2
SSI	46	0 (0)	0 (0)	
SSO/I requiring procedural intervention	46	0 (0)	0 (0)	
Reoperation	46	0 (0)	0 (0)	

ªData are presented as number (percentage). *N* reflects the number of patients with available data at a given time point. *SSO* Surgical site occurrence. *SSI* Surgical site infection

Table 5 demonstrates quality of life and recurrence outcomes. At 30 d, PROMIS Pain Interference scores were similar between groups (*p* = 0.4) and this trend continued at 1 year [(39 ± 9) vs (37 ± 8), *p* = 0.1]. The change in PROMIS Pain Interference score from baseline to 30 d was small and not statistically significant (*p* = 0.3), and it remained low and non-significant at 1 year (*p* = 0.6). HerQLes scores at 30 d were statistically similar, as was the mean improvement in HerQLes score at 30 d. At 1 year, both 1-year HerQLes scores and changes in HerQLes scores from baseline were similar between both groups and not statistically significant (*p* = 0.7 and 1) (Table 5).

**Table 5:** Quality-of-life outcomesª

Outcomes	*N*	NSGM (*n* = 689)	SGM (*n* = 689)	*p* value
HerQLes score				
30 days	466	65 ± 28	66 ± 24	0.9
Change from baseline	282	10 ± 30	14 ± 28	0.2
1 year	144	82 ± 21	79 ± 25	0.7
Change from baseline	68	22 ± 22	22 ± 28	1
PROMIS pain T score				
30 days	479	44 ± 10	44 ± 8	0.4
Change from baseline	291	0.5 ± 11.7	−0.9 ± 10.7	0.3
1 year	146	37 ± 8	39 ± 9	0.1
Change from baseline	68	−5 ± 9	−4 ± 8	0.6
Surgeon-reported recurrence				
1 year	41	1 (4)	0 (0)	0.5
2 years	11	0 (0)	1 (17)	0.3
3 years	3	0 (0)	0 (0)	NS
4 years	2	0 (0)	0 (0)	NS
5 years	1	NR	0 (0)	NS
Patient-reported recurrence				
1 year	147	9 (12)	14 (19)	0.3
2 years	152	2 (3)	18 (22)	< 0.001
3 years	97	5 (10)	12 (26)	0.04
4 years	62	4 (14)	14 (41)	0.02
5 years	40	2 (10)	6 (30)	0.1
Pragmatic recurrence				
1 year	275	8 (5)	13 (10)	0.2
2 years	202	2 (2)	18 (18)	< 0.001
3 years	131	5 (7)	12 (19)	0.05
4 years	85	4 (10)	14 (33)	0.009
5 years	50	2 (8)	6 (24)	0.1

^a^Data are presented as mean ± SD or number (percentage). *N* reflects the number of patients with available data at a given time point. *NR* None recorded, indicating that there were no instances of follow-up at that time point. *NS* Non-significant, with insufficient sample size for statistical comparisons

At 1 year, patient-reported recurrence rates trended higher in the SGM group (19 vs 12%, *p* = 0.3), with significantly higher rates of patient-reported recurrence in years 2–4. At year 5, SGM had a 30% rate of patient-reported recurrence compared to 10% for NSGM; however, this was not statistically significant, likely secondary to low rates of follow-up at that time point. In general, there were low rates of clinical follow-up, particularly past 1 year. At no point was there a statistically significant difference in surgeon-reported recurrence between groups. At 1 year, the rate of pragmatic hernia recurrence was numerically, though not statistically, higher in the SGM group (10 vs 5%, *p* = 0.2). The pragmatic recurrence rate was significantly higher in the SGM group at 2–4 years, becoming non-significant again at year 5.

In a subgroup analysis comparing SGM without additional fixation to NSGM with any form of fixation, 3081 patients were included. After PSM, 1064 remained (532 SGM and 532 NSGM). Operative times were similar between both groups (*p* = 0.4).

Table 6 demonstrates clinical outcomes of this subgroup. Early wound complications were infrequent in both groups. SSO at 30 d was similar between groups (5 vs 6%, *p* = 0.4). 30-day SSI, SSOPI, and reoperation were rare and similar between groups (*p* = 0.7, 0.5, and 1 respectively). At 1 year, SSI and SSOPI remained rare and comparable between both groups (0 vs 0%). Although SSO rates trended higher in the SGM group at 1 year (7 vs 0%, *p* = 0.2), this difference was not statistically significant.

**Table 6:** Post-operative outcomes after subgroup analysesª

Outcome	*N*	NSGM (*n* = 532)	SGM (*n* = 532)	*p*-value
30-day follow-up				
SSO	1064	27 (5)	34 (6)	0.4
SSI	1064	3 (1)	2 (0)	0.7
SSO/I requiring procedural intervention	1064	6 (1)	4 (1)	0.5
Reoperation	1063	4 (1)	4 (1)	1
1-year follow-up				
SSO	39	0 (0)	1 (7)	0.2
SSI	39	0 (0)	0 (0)	1
SSO/I requiring procedural intervention	39	0 (0)	0 (0)	1
Reoperation	39	0 (0)	0 (0)	1

ªData are presented as number (percentage). *N* reflects the number of patients with available data at a given time point. *SSO *Surgical site occurrence. *SSI* Surgical site infection

Table 7 demonstrates quality of life and recurrence outcomes for this subgroup. PROMIS and HerQLes scores were similar between groups at 30 d and 1 year. Surgeon-reported recurrence was rare for both groups at all time points. Patient-reported recurrence was significantly higher in the SGM group between years 2 and 4. Pragmatic recurrence at 1 year was statistically similar between groups (13 vs 6%, *p* = 0.06); however, at years 2–4, rates of pragmatic hernia recurrence were significantly higher in the SGM group.

**Table 7:** Quality-of-life and recurrence outcomes after subgroup analysesª

Outcomes	*N*	NSGM (*n* = 532)	SGM (*n* = 532)	*p* value
HerQLes score				
30 days	356	68 ± 26	66 ± 24	0.3
Change from baseline	216	10 ± 29	14 ± 28	0.2
1 year	131	84 ± 18	78 ± 25	0.5
Change from baseline	65	26 ± 25	21 ± 30	0.7
PROMIS pain T SCORE				
30 days	367	44 ± 9	44 ± 8	0.9
Change from baseline	224	1 ± 11	-1 ± 11	0.06
1 year	133	36 ± 8	39 ± 9	0.07
Change from baseline	66	−7 ± 11	−4 ± 8	0.2
Surgeon-reported recurrence				
1 year	36	0 (0)	0 (0)	NS
2 years	13	0 (0)	1 (17)	0.3
3 years	3	0 (0)	0 (0)	NS
4 years	2	0 (0)	0 (0)	NS
5 years	3	1 (50)	0 (0)	0.4
Patient-reported recurrence				
1 year	134	9 (12)	14 (23)	0.09
2 years	138	5 (7)	16 (24)	0.006
3 years	79	5 (12)	11 (30)	0.05
4 years	54	5 (19)	12 (44)	0.04
5 years	35	2 (11)	5 (31)	0.1
Pragmatic recurrence				
1 year	234	8 (6)	13 (13)	0.06
2 years	174	5 (5)	16 (20)	0.003
3 years	105	5 (9)	11 (23)	0.04
4 years	73	5 (12)	12 (38)	0.01
5 years	44	3 (12)	5 (28)	0.2

ªData are presented as mean ± SD or number (percentage). *N* reflects the number of patients with available data at a given time point

## Discussion

To our knowledge, this is the first comparative analysis of SGM versus NSGM in MIS extraperitoneal VHR. Despite the theoretical advantages of SGM related to reduced fixation requirements and the potential for decreased postoperative pain, we did not identify any clinically or statistically significant benefit of SGM over NSGM. These findings persisted in subgroup comparisons specifically designed to demonstrate the greatest potential benefits of SGM. Exploratory long-term analyses suggested higher rates of hernia recurrence in the SGM cohort. Collectively, these data challenge use of SGM in MIS extraperitoneal VHR.

The absence of short-term wound morbidity benefits with SGM may be explained by the operative context. In open VHR, baseline rates of wound morbidity are typically higher than with minimally invasive VHR [15]. It is thought that some of the benefit of SGM over NSGM in the context of open repair may stem from transfascial suture fixation of NSGM acting as a nidus for infection. Given recent randomized controlled trial literature around transfascial fixation and its lack of superiority over non-fixation, it is possible that this apparent clinical benefit will be seen less frequently as transfascial fixation is abandoned for many patients [16]. In MIS extraperitoneal repairs, baseline wound morbidity is already substantially reduced due to smaller incisions [1]. Additionally, mesh fixation used in this context is typically not transfascial and is often limited to 4–5 locations, which may reduce potential benefits seen with SGM around wound morbidity, pain, and quality of life. The subgroup analysis performed in this study was specifically designed to highlight any potential pain or quality of life benefit secondary to use of SGM without fixation in comparison to NSGM with fixation. No such benefit was observed. The routine selection of SGM in MIS extraperitoneal VHR for anticipated reduction in postoperative pain does not seem to be justified. Surgeons should recognize that in extraperitoneal repairs, mesh fixation strategy may play a smaller role in early pain and quality of life outcomes than previously thought.

There was no observed benefit of SGM regarding operative time. In fact, operative times were slightly longer in the SGM group. SGM used in the minimally invasive context requires time spent to ensure that the mesh is well opposed to the abdominal wall as insufflation is reduced. It is likely that the time spent doing this is similar to, if not greater than, the time commonly spent to place 4–5 interrupted sutures when fixation is used with NSGM. One limitation of ACHQC data is that operative times are collected in 60-min intervals, decreasing the precision with which time comparisons may be made. Nevertheless, if a clinically significant and consistent benefit were to exist, it would be expected to shift the distribution of categorical operative times.

Exploratory long-term analysis suggested a higher rate of pragmatic hernia recurrence with SGM in this context. This warrants careful interpretation as these outcomes were significantly reliant on patient-reported outcomes and long-term follow-up was limited in comparison to the overall cohort. At 1 year, for instance, recurrence data were only available for 20% of the study population. Nevertheless, a consistent trend over the available long-term follow-up was seen, and consideration as to why this might occur is necessary. Our findings differ from some prior reports suggesting low rates of long-term recurrence with SGM in open sublay repair [4, 17, 18]. One potential factor would be the degree of mesh overlap. The degree of craniocaudal mesh overlap was similar between groups in this study; however, the degree of lateral mesh overlap was lower in the SGM group, with mean lateral overlap of 4 cm compared to 5 cm in the NSGM group. The current European Hernia Society guidelines recommend mesh overlap of at least 5 cm or more in laparoendoscopic repairs to decrease recurrence rates, and therefore the fact that many patients in the SGM group did not achieve this may have contributed in part to these recurrence outcomes. It is unclear why overlap would differ, though it may be that available sizes of SGM presented constraints in terms of achieving overlap or that surgeons were trying to use slightly smaller sizes of SGM, potentially related to cost.

Another potential technical mechanism leading to the observed recurrence rates in this study may be secondary to the orientation of the mesh. In an open context, the grips of the mesh may be oriented down toward the peritoneum, allowing the bare mesh to have improved contact with the repaired muscle/fascia of the abdominal wall. In an MIS extraperitoneal fashion, grips are likely deployed facing the muscle/fascia, with bare mesh apposing peritoneum and/or posterior rectus sheath. It is unclear whether this difference in orientation may impact early mesh ingrowth in clinical practice and potentially contribute to the long-term recurrence differences noted in this study. Additionally, the insufflation pressure at which SGM is placed may influence whether it remains appropriately adherent to the abdominal wall or becomes wrinkled as insufflation is decreased. The selection of SGM on the market is limited, and it is possible that mesh weight or material may have played into these outcomes as well, as only polyester-based SGM were available during the study period. These are factors that were not analyzed in the present study but which will merit further study as more SGM are introduced. Given the observed recurrence trend, careful attention to mesh overlap, defect closure, and patient selection is essential if considering SGM in this context. These findings underscore the importance of structured postoperative surveillance beyond the early postoperative window, as clinically meaningful differences in recurrence may not manifest until years after surgery.

In seeking high-value care for patients, the aim is for high-quality outcomes for patients at low costs. Quality in the context of hernia surgery can be conceptualized as including not only hernia recurrence, but also operative time, length of stay, wound morbidity, pain, and quality of life [19]. In the present analysis, SGM did not seem to offer higher-quality short-term outcomes compared to NSGM, but instead demonstrated higher long-term pragmatic recurrence rates. This, in and of itself, is concerning and would call into question the use of SGM in this context. Financial cost to patients and health care systems must also be considered. This will vary between systems, but frequently, SGM will cost more than NSGM alternatives. In our own system, a 15 × 15 cm SGM costs 2.4 times as much as a similarly weighted, uncoated NSGM of the same size. Based on the present results, NSGM appears to offer greater value to patients and health care systems than SGM in the context of MIS extraperitoneal VHR.

This study has several strengths. It draws from a large, prospectively maintained international database, and therefore reflects real-world practice and outcomes. Evaluation of both short-term and longer-term outcomes provides a comprehensive assessment of outcomes with these prostheses. A robust number of patients were included, increasing the validity of the early postoperative outcomes analyzed.

Nevertheless, this study has limitations. The retrospective design introduces potential selection bias as well as the possibility of unmeasured confounders. Despite propensity score matching to reduce confounding, this study cannot prove causation, only association. Our primary outcome was at the 30-day time point where clinical follow-up data were complete, and we have noted that the longer-term outcomes are exploratory. Long-term in-person follow-up was limited, which is common within the retrospective hernia literature. Patient-reported recurrences may not always reflect clinical recurrences; however, the distribution of patient-reported recurrences would not be expected to differ between two interventions absent some mediating factor. Likewise, whether patients perceive themselves to have a hernia or bulge after a hernia intervention is important to patients, even if their perception does not align with objective measures of recurrence.

In conclusion, in MIS extraperitoneal VHR, SGM did not demonstrate measurable benefits over NSGM in short-term perioperative and patient-reported outcomes. A persistent signal toward higher long-term hernia recurrence with SGM warrants further close study and surgeons who utilize SGM in this context should actively monitor their long-term recurrence rates.
